# Œdème papillaire bilatéral secondaire à une hypertension intracrânienne chez une adolescente

**DOI:** 10.11604/pamj.2017.26.123.11463

**Published:** 2017-03-03

**Authors:** Seydou Bakayoko, Nouhoum Guirou, Brainima Coulibaly, Jeannette Traoré

**Affiliations:** 1 Centre Hospitalier Universitaire, Institut d'Ophtalmologie Tropicale d'Afrique, Boulevard du Peuple, Bamako, Mali, Boulevard du Peuple, Bamako, Mali

**Keywords:** Œdème, papille, angiographie, Edema, papilla, angiography

## Abstract

L'œdème papillaire est un gonflement liquidien et/ou axonal de la tête du nerf optique du à un blocage du flux axoplasmique au niveau de la lame criblée. Nous rapportons le cas d'une jeune adolescente âgée de 17 ans, qui présenta un œdème papillaire bilatéral secondaire à une hypertension intracrânienne idiopathique.

## Abstract (en)

Papilledema is a fluid and/or axonal swelling of the optic nerve head due to a blockage of axoplasmic flow at the level of the cribriform plate. We report the case of a 17-year old adolescent girl with bilateral papilledema secondary to idiopathic intracranial hypertension.

## Introduction

L'œdème papillaire est l'expression clinique d'affections diverses. Il s'agit d'un gonflement liquidien et/ou axonal de la tête du nerf optique du à un blocage du flux axoplasmique au niveau de la lame criblée [1, 2].

## Patient et observation

**Figure 1:**
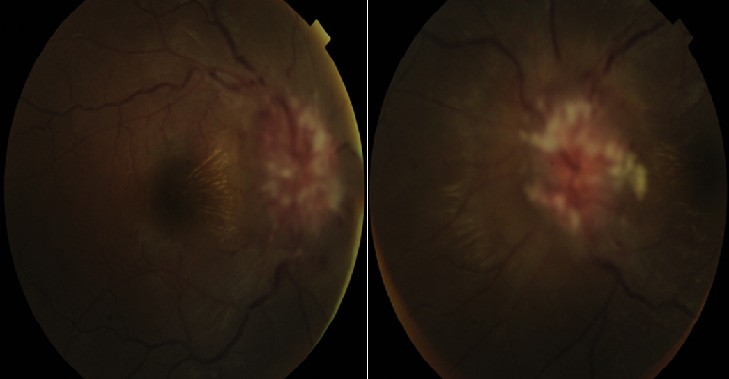
Cliché couleur

**Figure 2:**
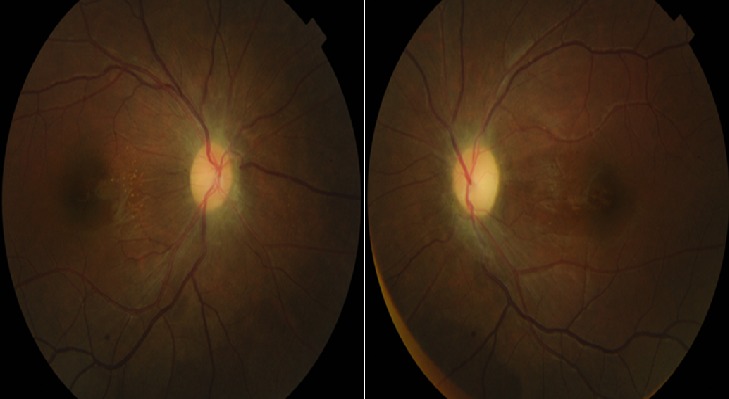
Cliché couleur

Mlle R F, âgée de 17 ans, admise dans notre service plus d' un mois après l'apparition de nausée, de céphalées et une baisse de la vision. Elle avait été vue en neurologie pour les mêmes symptômes. L'état général était satisfaisant, sans variation pondérale récente ni surcharge. A l'interrogatoire, aucune prise médicamenteuse ni vaccination n'étaient signalée. L'examen neurologique avec tomodensitométrie cérébrale ne révélait aucune anomalie. A l'examen ophtalmologique, l'acuité visuelle était réduite à 1/20^ème^ sur chaque œil avec un segment antérieur normal. La pression intra oculaire était de 17 mm Hg. Au fond d'œil, il existait un œdème papillaire bilatéral avec des nodules cotonneux, des hémorragies et un aspect exsudatif d'étoile maculaire Figure 1. Le champ visuel était perturbé de façon globale et non systématisé avec élargissement de la tache aveugle. L´angiographie a confirmé l'œdème par une hyperfluorescence papillaire. La ponction lombaire a montré un LCR hypertendu de composition normale, évocateur d'un syndrome d'HTIC bénigne. Les examens biologiques (sérologie syphilitique TPHA/VDRL, Toxoplasmique IgG/IgM) étaient négatifs. Un traitement par acétazolamide 250mg a été prescrit à la dose de 375 mg/jour et une corticothérapie pendant 10 jours ont permis l'amélioration de l'acuité visuelle (10/10^ème^ faible œil droit et 9/10^ème^œil gauche) 6 semaines plutard mais avec pâleur modérée de la papille Figure 2. La normalisation du champ visuel a été obtenue au 2^ème^ mois.

## Discussion

Il s'agit d'un œdème papillaire de stase bilatéral idiopathique ou pseudotumor cerebri dont la symptomatologie fonctionnelle était marquée par des céphalées, des nausées et une baisse sévère de l´acuité visuelle. Les manifestations cliniques de l'HICB sont celles de toute HIC. Les céphalées avec nausées et/ou vomissements constituent l´une des manifestations initiales majeures dans la plupart des séries comme dans notre cas [3, 4]. L´acuité visuelle est habituellement conservée pour les œdèmes papillaires de constitution récente [1, 2, 5]. L'œdème papillaire peut être associé à une baisse de l'acuité visuelle comme dans notre cas; cette atteinte de l'acuité a été notée par d'autres auteurs ; une atteinte transitoire dans 50 à 60% [3] et de 10 à 30% [4]. Habituellement l'association d'un œdème papillaire et une étoile maculaire (partiel ou total) définit le tableau clinique d'une neurorétinite [6] dont les étiologies sont multiples, incluant principalement des pathologies infectieuses, inflammatoires locales ou systémiques mais aussi des atteintes vasculaires. Dans notre cas nous éliminé cette hypothèse devant le jeune âge de notre patiente et en absence de foyer de rétinite ou de choroïdite au fond d'œil. Nous avons donc retenu le diagnostic d'hypertension intracrânienne bénigne. L' hypertension intracrânienne bénigne est définie par les critères diagnostiques suivants : présence de céphalées, absence de signe neurologique de focalisation, normalité de l'examen neuroradiologique, normalité de la biologie du liquide céphalorachidien avec augmentation de la pression à la ponction [1, 5, 7, 8]. Dans notre cas la patiente a été mise sous Acetazolamide (Diamox) associé à une corticothérapie de courte durée. Nous avons obtenu une remontée de l'acuité visuelle en 10 jours sous acétazolamide seul ; cela pourrait s'expliqué par le fait que cette HIC était probablement transitoire. D'autres auteurs [5, 7, 9, 10] ont prouvé l'efficacité du traitement par Acetazolamide seul ou parfois associée à une corticothérapie selon les cas.

## Conclusion

Devant unœdème papillaire bilatéral, le diagnostic d´HTIC bénigne idiopathique doit rester un diagnostic d´élimination. Le diagnostic, comme le traitement peut s´avérer multidisciplinaire associant ophtalmologiste, radiologue et neurologue et neurochirurgien.
